# The distribution of insertionally polymorphic endogenous retroviruses in breast cancer patients and cancer-free controls

**DOI:** 10.1186/s12977-014-0062-3

**Published:** 2014-08-12

**Authors:** Julia H Wildschutte, Daniel Ram, Ravi Subramanian, Victoria L Stevens, John M Coffin

**Affiliations:** Department of Molecular Biology and Microbiology, Tufts University School of Medicine, 136 Harrison Avenue, Boston, MA 02111 USA; Department of Immunology, Tufts University School of Medicine, Boston, MA 02111 USA; Epidemiology Research Program, American Cancer Society, Atlanta, GA 30303 USA; Present address: Department of Human Genetics, The University of Michigan Medical School, 1241 E. Catherine St, Ann Arbor, MI 48109 USA

**Keywords:** Endogenous retrovirus, Provirus, HERV-K, Breast cancer, Betaretroviridae, MMTV, JSRV

## Abstract

**Background:**

Integration of retroviral DNA into a germ cell can result in a provirus that is transmitted vertically to the host’s offspring. In humans, such endogenous retroviruses (HERVs) comprise >8% of the genome. The HERV-K(HML-2) proviruses consist of ~90 elements related to mouse mammary tumor virus, which causes breast cancer in mice. A subset of HERV-K(HML-2) proviruses has some or all genes intact, and even encodes functional proteins, though a replication competent copy has yet to be observed. More than 10% of HML-2 proviruses are human-specific, having integrated subsequent to the *Homo-Pan* divergence, and, of these, 11 are currently known to be polymorphic in integration site with variable frequencies among individuals. Increased expression of the most recent HML-2 proviruses has been observed in tissues and cell lines from several types of cancer, including breast cancer, for which expression may provide a meaningful marker of the disease.

**Results:**

In this study, we performed a case–control analysis to investigate the possible relationship between the genome-wide presence of individual polymorphic HML-2 proviruses with the occurrence of breast cancer. For this purpose, we screened 50 genomic DNA samples from individuals diagnosed with breast cancer or without history of the disease (n = 25 per group) utilizing a combination of locus-specific PCR screening, *in silico* analysis of HML-2 content within the reference human genome sequence, and high-resolution genomic hybridization in semi-dried agarose. By implementing this strategy, we were able to analyze the distribution of both annotated and previously undescribed polymorphic HML-2 proviruses within our sample set, and to assess their possible association with disease outcome.

**Conclusions:**

In a case–control analysis of 50 humans with regard to breast cancer diagnosis, we found no significant difference in the prevalence of proviruses between groups, suggesting common polymorphic HML-2 proviruses are not associated with breast cancer. Our findings indicate a higher level of putatively novel HML-2 sites within the population, providing support for additional recent insertion events, implying ongoing, yet rare, activities. These findings do not rule out either the possibility of involvement of such proviruses in a subset of breast cancers, or their possible utility as tissue-specific markers of disease.

## Background

Breast cancer is the most common cancer and second most common fatal cancer among women in the United States. In 2014, according to American Cancer Society (ACS) estimates, 232,670 women will have been diagnosed with breast cancer and at least 40,000 women will have died from this malignancy in the United States [1]. It is the leading cause of cancer-related death in women of Caucasian, African-American, Asian, and Native American ethnicities, and is the most common cause of death in Hispanic women. However, the incidence of breast cancer varies with respect to ethnic populations, suggesting underlying genetic, environmental, or lifestyle influences in its development and/or progression [1,2].

In recent years there have been significant discoveries that have contributed to improved prevention and diagnosis of breast cancer. Most notable are the discoveries of the *BRCA1* and *BRCA2* genes, identified in multiple-case family studies in which breast cancer cases were observed to follow a Mendelian pattern of inheritance [3–5]. Subsequent family-based studies have failed to identify additional genes associated with increased breast cancer risk, although *BRCA1* and *BRCA2* account for just 20 to 40% of familial cancers and about 5% of all breast cancer cases worldwide [6]. More recently, large-scale genotyping and genome-wide association (GWA) studies have led to the identification of other breast cancer susceptibility loci [5,7–9], which are currently estimated to account for less than 2 to 10% of disease risk, leaving at least 50% of breast cancer risk that remains to be explained [10]. Although GWA studies have expanded key areas of breast cancer research, their nature is inherently self-limiting due to reliance on single nucleotide polymorphisms (SNPs). As a result, other sources and types genomic and structural variation -that are also polymorphic and inherited in Mendelian fashion- are excluded, including recently mobile genetic retroelements, leaving the possibility of their disease association closed to investigation in such analyses.

More than 8% of the human genome is recognizably of retroviral origin, representing the remnants of ancient germline infections from exogenous retroviruses [11]. During an active retroviral infection cycle, the viral genomic RNA is reverse transcribed into a double-stranded DNA copy that is then permanently integrated into the host genome. Thus, the integration of retroviral DNA into a germ line cell may lead to a provirus that is transmitted vertically to that host’s offspring as an endogenous retrovirus (ERV) [12]. If such an integration event has no immediate negative affect to the host, the provirus may be passed successively from parent to offspring over generations, eventually gaining population-wide polymorphic persistence and even fixation within the population. The vast majority of human ERVs (HERVs) were formed from germline infection and integration tens of millions of years ago, having since become highly mutated and truncated, or recombined to form solo LTRs, and are thus present without any infectious or functional capacity. However, a small number of HERVs -particularly those having formed within the last few million years– have retained at least some coding capacity and many are actively transcribed in certain cancers as well as some normal tissues [13,14].

The most recent retroviruses to colonize the human germ line are from the betaretrovirus-like HERV-K(HML-2) group, most closely related to the exogenous mouse mammary tumor virus (MMTV) and Jaagsiekte sheep retrovirus (JSRV), which respectively cause breast cancer in mice and lung cancer in sheep [15–20]. Within the human genome, the HML-2 group of proviruses is represented by approximately 90 proviruses and about 1000 solitary LTRs [19]. Unique among HERVs, the HML-2 group includes at least 23 human-specific proviruses, of which 11 are currently known to have polymorphic alleles of varying frequency within the population [16,17,19,20]. Genome-wide and population-based screens have provided a strong indication for the presence of other unique, polymorphic HML-2 proviruses within some humans, and additional insertions are likely to be identified in the near-future with improved genome sequencing technologies and population-wide detection strategies; however, research into the patterns and prevalence of such HERVs is lacking [17,21]. The possibility remains that members of this group are still capable of replication, either from very rare but still-active individual proviruses or from the formation of a replication-competent recombinant via complementation of expressed and co-packaged viral RNAs into a budding particle. In support of this possibility, most human-specific and all polymorphic HML-2 proviruses have more than one intact open reading frame (ORF), and some encode functional proteins and even retrovirus-like particles (RVLPs) [19,22–27]. Also, the rate of accumulation of HML-2 proviruses in the human genome appears to have been constant since the *Homo-Pan* divergence [21]. Although a naturally occurring HML-2 provirus with infectious capacity has yet to be observed, engineered consensus HML-2 proviruses are weakly infectious [28,29].

A growing number of reports continues to demonstrate increased levels of HML-2 transcripts and proteins in affected tissues from several types human disease, including but not limited to ovarian cancer [30], germ cell tumors [24,31–34], melanomas [35–40], and leukemias/lymphomas [41,42]. Of particular interest has been HML-2 proviral expression in diseased tissues associated with breast cancer, with up-regulation of HML-2 both from breast tumor biopsies and cell lines derived from breast tumor tissues [41,43–48]. In matched-tissue analyses, spliced and unspliced HML-2 *env* transcripts have been detected in cancerous breast tissue, but not adjacent normal epithelia [43,46,47]. Also, the release of HML-2-encoded RVLPs associated with encapsidated, unspliced transcripts and RT activity has been shown for multiple breast cancer-derived cell lines [49–51]. While the consequence of endogenous HERV expression is poorly understood, an essential relationship may be inferred through the genetic association of an inherited provirus to a particular disease, as is known to occur in a few animal models, such as the association of certain MMTV proviruses and mammary carcinoma in mice [52,53]. Given their variable presence within the population and high levels of functional conservation, only the HML-2 group of HERVs contains representative candidates for such a scenario.

Two HML-2 proviruses, referred to as K113 and K115 (located respectively at chromosomal regions 19p12 and 8p23.1) have been examined for possible disease association [54–57]. Present respectively within ~30% and ~15% of individuals tested, K113 and K115 are estimated to have integrated into the germline <2mya and have functional ORFs [19,20,57]. At least one report has investigated the prevalence of K113 and K115 among breast cancer patients [54], however the prevalence of other polymorphic HML-2 proviruses has not been addressed. Furthermore, the presence of additional unique yet currently uncharacterized polymorphic HML-2 proviruses within the population [17] makes it difficult to conclusively test for a genetic association using conventional methods, such as microarray hybridization or genomic sequencing, which are essentially blind to the detection such unannotated genomic variation.

We report the distribution of polymorphic HML-2 proviruses, including elements not previously characterized, in a cohort of breast cancer patients and individuals with no history of the disease. In a combined approach using PCR screening and ‘unblotting’ , or direct hybridization of DNA within semi-dried agarose, a high-resolution technique previously developed and used by our lab to characterize endogenous murine leukemia viruses [14,58], we investigated the prevalence of individual polymorphic HML-2 proviruses in a case–control comparison. Although we found no significant difference in the prevalence of individual proviruses between groups, suggesting that common polymorphic HML-2 proviruses (present in >5% individuals tested) are not associated with breast cancer. However, these findings do not exclude either the possibility that rarer HML-2 proviruses are somehow involved in a subset of breast cancers or will provide a meaningful biomarker of this disease.

## Results

### Analysis of annotated polymorphic HML-2 proviruses in breast cancer patients

We first sought to examine the prevalence of the currently described polymorphic HML-2 proviruses in a case–control analysis in order to determine whether any was detected with a strong difference in frequency between groups, and to provide a direct comparison for the subsequent analysis of previously uncharacterized polymorphic proviruses. For these purposes, we screened a panel of genomic DNA samples from diagnosed breast cancer patients and individuals with no history of the disease. Samples were generously provided by the American Cancer Society (ACS) and were from the Cancer Prevention Study II Nutrition Cohort (CPS-II). CPS-II is a large-scale study designed to provide a prospective means for investigating the relationship between lifestyle factors and exposure risk to cancer incidence, mortality, and survival [59]. We initially analyzed 50 unlinked and de-identified genomic DNA samples from breast cancer cases or controls (n = 25 per group).

Previous work from our lab and by others has led to the identification of 11 examples of HML-2 proviruses for which multiple alleles can be detected with varying frequencies among humans (Table 1) [15–17,19,20]. We verified the chromosomal locations for 8 of the 11 polymorphic proviruses within the February 2009 human genome build (GRCh37/Hg19), with reference to parallel BLAT searches against earlier genome builds (March 2006 Hg18; May 2004 Hg17; July 2003 Hg16). For a conventional and consistent nomenclature reference [19], the proviruses included here are identified by their chromosome location and position relative to other proviruses if multiple proviruses are present within the same chromosomal band. The full-length sequences of four elements are absent from all published builds: two proviruses, located at 10p12.1 (also referred to as K103) and at 12q13.2, are represented as solo LTRs; the 19p12b (K113) insertion site is empty, with no evidence of a polymorphic provirus at the site; the remaining provirus (referred to as K105) is integrated within the unassembled centromeric region Un_g1000219 and unaligned to the current genome build. However, the genomic regions flanking each integration site are publicly available (respectively JN675098.1, JN675106.1, JN675117.1, and JN675176) [19], and BLAT searches were performed to verify each chromosomal location.

**Table 1:** **Known polymorphic HML-2 proviruses in human DNA**

**HERV-K notation**	**Locus**	**Start (bp) in Hg19**	**Alleles** ^***b***^	**Accession number**	**Reference**
	1p31.1	75842771	pro	AC093156.2	[16]
*K106*	3q13.2	112743479	pro, solo	AC024108.22	[14]
*K109*	6q14.2	78427019	pro, solo	AC164615.1	[14,16]
*K108* ^*a*^	7p22.1^*a*^	4630561	pro, solo, tandem, pre	AC164614.1	[16,26]
*K115*	8p23.1	8054700	pro, pre	AY037929.1	[19]
*K103*	10p12.1	27182399	pro, solo	AF164611.1	[14]
	11q22.1	101565794	pro, solo, pre	AP000776.5	[16,25]
	12q13.2	55727215	pro, solo, pre	JN675067	[18,20]
	12q14.1	58721242	pro, solo	AC074261.3	[16,25]
*K113*	19p12	21841536	pro, pre	AY037928.1	[19]

^*a*^K108 is present as a tandem provirus in the published genome with a single shared LTR in the middle. The start coordinate refers to the right provirus of the tandem pair.

^*b*^Pro, provirus; solo, solo LTR; pre, pre-integration (empty) site.

Initial HML-2-specific PCR screening was performed with all CPS-II samples blinded and randomly sorted. Locus-specific amplification was performed to detect the alleles present at each HML-2 insertion site, with primers spanning either the 5’ LTR of each provirus (indicating the presence of the more or less full-length allele) and spanning the integration site (to detect either a solo LTR or the ancestral pre-integration sequence) (Table 2). Representative products from each amplified site were sequenced in both directions to confirm the correct product and to ensure primer specificity (data not shown). Upon completion of the primary screen, information for the disease group (breast or prostate cancer) and case/control identity was unblinded, and the samples sorted and grouped accordingly. PCR amplification for each HML-2 integration site was repeated as above to confirm the initial results, and to provide a direct case–control comparison for the breast cancer sample group. The frequency of each provirus was calculated per site per group, and the results subjected to a χ^2^ analysis, with a *p*-value of <0.05 regarded as significant within the dataset. The results are summarized in Table 3.

**Table 2:** **Primers and product sizes for the detection of polymorphic HML-2 proviruses**

**Locus** ^***a***^ ***(synonym)***	**Forward (5’➔3’)**	**Reverse (5’➔3’)**	**Size (bp)** ^***b***^ **solo/pre**	**Predicted** ***Bsr*** **1 Fragment size (bp)**
1p31.1-I	AACTACGTGAAGAATGAAGA	AATAAAGCTGAGATAAGAGG	1239	1752
3q13.2-I	GCTCGGATTTCAACATCCAT	TCGTCCGACTTGTCCTCAATG	1821	1985
3q13.2-II	GCTCGGATTTCAACATCCAT	TATTGGTGACAGAGAGATGCAG	1847/879	
6q14.1-I	TCGTCGACTTGTCCTCAATG	CTGCCAGTCTCAGGTGTTTG	1075	1758
6q14.1-II	CCCCTGCTTATTGATGCTCTACG	TGAGGCTGAATGTGTGGAGTCC	1526/556	
7p22.1a-I	TACTGAACGATGCTGACGTTTGG	TTTGAACCATTATCACCCTA	1407	2067
7p22.1b-T	GTCTGCAGGTGTACCCAACAG	TTTGCCCCATTATCACCCTA	1216	1981
7p22.1-II	CCTCCTGGTTCAAGGGATTCTC	GCTTTCGGGACTTCAACATTGG	1387/419	
8p23.1a-I	CTTGTGTTTTCATTACAATCTATT	TTCAGTCATTCTATCATTAAGATTC	1667	2513
8p23.1a-II	CAGTCTATAGATGTGGATGCCT	AGCACTGAATCCAAACTCATAT	1320/352	
10p12.1-I	CCACCATCTGAGAAGTGTGATG	AATGGAGTCTCCYATGTCTACT	1342	1846
10p12.1-II	CCACCATCTGAGAAGTGTGATG	GGCAACAAAGGGTTCATATGAGAA	1508/540	
11q22.1-I	CCATGCTCAGAAAGGAAACA	TAGCTTCTTCCGAGCACACA	1168	2076
11q22.1-II	CCATGCTCAGAAAGGAAACA	ACCATCTGTCCTTCCACCAG	1661/693	
12q13.2-I	CGGAGAATTCCACCTTCAAA	CTCGAGCGTACCTTCACCCTAG	1377	2392
12q13.2-II	CGGAGAATTCCACCTTCAAA	TGCATTGTGGTCATCCATTT	1488/520	
12q14.1-I	GGAAACCCTTCCAACATTCCA	CCCCATTATCACCCTAGCTTC	1299	1755
12q14.1-II	GGAAACCCTTCCAACATTCCA	TGAGGCTGAATGTGTGGAGTCC	1101/133	
19p12b-I	TGCATGGGGAGATTCAGAACC	TCGGGATCTCTCGTCGACTTGTCC	1210	5287
19p12b-II	TGCATGGGGAGATTCAGAACC	CGTGTTAGCCAGGATGGTCT	310/1278	

^*a*^‘I’ specifies primers for the 5’LTR; ‘II’ specifies primers for either the solo LTR or empty site.

^*b*^Product sizes were estimated using *in silico* PCR (UCSC Genome Browser) of primer pairs. Product sizes for alleles for the 10p12.1, 12q13.2, and 19p12b proviruses were estimated manually by adding the distances to the distance to the nearest BsrI site in the host genome regions flanking each integration site and in the respective provirus for that site.

**Table 3:** **Prevalence of polymorphic HML-2 proviruses in breast cancer**

**HML-2 Locus**	**Breast cancer cases** ^***a***^		**Healthy controls** ^***a***^			
	**# Positive**	**Frequency**	**# Positive**	**Frequency**	**χ** ^**2**^	***p*** **-value** ^***b***^
*1p31.1*	16	0.64	17	0.68	0.09	0.76
*3q13.2*	25	1.00	25	1.00		
*6q14.2*	21	0.84	23	0.92	0.75	0.34
*7p22.1R*	25	1.00	25	1.00		
*7p22.1 L*	24	0.96	25	1.00	1.02	0.31
*8p23.1a*	6	0.24	1	0.04	4.15	0.04*
*10p12.1*	24	0.96	25	1.00	1.02	0.31
*11q22.1*	23	0.92	20	0.80	1.49	0.22
*12q13.2*	20	0.80	21	0.84	0.13	0.72
*12q14.1*	23	0.92	22	0.88	0.22	0.67
*19p12b*	3	0.12	3	0.12		

^*a*^Band sizes are based on estimated fragment lengths; each has been indicated by arrow in Figure 2.

^*b*^Total sample size was 50 (n = 25 per group).

*Indicates significance (p > 0.05) within the dataset (not corrected for multiple comparisons).

The majority of HML-2 insertion sites examined had no significant difference in proviral frequencies between groups. However from our initial case–control screens, we observed the K115 provirus to be present at a higher prevalence within breast cancer cases (6/25, or to a frequency of 0.24) than in the control group (1/25, or 0.04), with a *p*-value of 0.04. On a preliminary basis, this observation was of interest, given the significant difference in frequency between groups for the sample size. However, this particular provirus has been previously analyzed for possible association with a few human diseases (including breast cancer [54]), without significant support. Thus, we attempted to test the observed difference within a larger collection of representative genomic samples (to >90% statistical power). For this purpose, a unique set of 200 CPS-II samples (100 breast cancer cases and 100 controls) was analyzed for the presence of K115 alone. We found that the initial result was not corroborated in the repeat analysis, in which K115 was observed in 6/100 cases (0.06) and 11/100 controls (0.11) (corresponding to a χ^2^ of 1.61 and *p*-value of 0.20). Collectively, these results suggest that no described individual polymorphic HML-2 provirus is associated with breast cancer occurrence for the CPS-II genomic samples screened; however these results do not exclude the possible association of HML-2 occurrence within a subset of breast cancer cases, or other disease types with implication for involvement.

Of the described polymorphic HML-2 proviruses, most are present in relatively high allele frequencies within humans (~50% or above), and even the K113 and K115 proviruses are present in as many as 30% to 40% of tested individuals, depending on the ethnicity (on average, within ~16-20% random individuals tested) [20,57]. Aside from the 11 described polymorphic integration sites, there is evidence that other unique polymorphic HML-2 proviruses are present in varying frequencies within humans [14,16,17,20]. However, the population distributions, genomic locations, and any sequence information of such elements are unknown. Previous work in our lab has shown that ERVs can be detected from fragmented genomic DNA by utilizing a high-specificity hybridization technique referred to as ‘unblotting’, during which restriction enzyme digested DNA is hybridized with a radiolabeled probe while immobilized in semi-dried agarose following electrophoresis [14,17,58]. Using this technique, polymorphic integrations can be identified as bands that vary between samples, and provides the means for direct comparison between individuals and/or groups. Therefore, we used unblotting to estimate the total number, distribution, frequency, and potential disease association of individual polymorphic HML-2 proviruses, including known integrations and those not previously described in the current genome databases, within our sample set.

### *In silico* analysis of polymorphic proviruses

Initially, we performed *in silico* analysis as a means both to identify appropriate restriction enzymes for unblot analysis, and to generate predicted fragment patterns of previously annotated HML-2 proviruses with reference to the published genome sequence. For these purposes, we mined the Hg19 genome build for proviruses with high nucleotide identity to HERV-K113 (19p12b). We chose this full-length provirus as a reference since it is completely intact and represents one of the most evolutionarily recent germline integrations [19,20]. Full-length sequences were extracted for a total of 62 identified proviruses, to which 5 other described proviruses (located at 10p12.1 (K103), 19p12b (K113), at 12q13.2, and the K105 provirus located within an unaligned contig, Un_gl000219 [19] were manually added.

**Figure 1:**
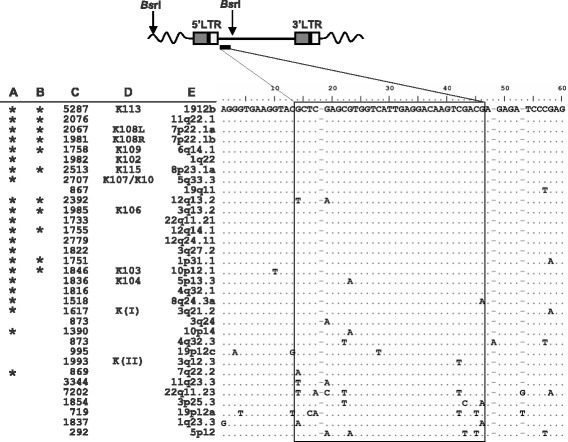
**Identification of a conserved sequence for the detection of recently integrated HML-2 proviruses.** A BLAT search of the 2009 human genome sequence build GRCh37/Hg19 for the ~32 bp K-seq sequence (shown in box) returned each provirus included in the alignment. The aligned sequences have been ordered with reference to percent identity to the K113 nucleotide sequence to depict the conservation of the region among the most recently formed germline integrations. Bases shared with K113 are indicated as dots, and differences are indicated by the base present at that site. Asterisks at left indicate **A**. human-specific and **B**. polymorphic proviruses; **C**. predicted fragment sizes (in bp) based on restriction analysis of the published human genome (Hg19); **D**. reference aliases of each provirus; **E**. chromosomal locus of each analyzed element.

To identify a suitable probe sequence, we aligned and manually edited the full nucleotide sequences of all 66 proviruses, sorted individual elements in the resulting alignment by decreasing nucleotide identity to K113, and searched the alignment for sequence regions that were 1) highly similar among the most recently integrated elements (*i.e.*, polymorphic and/or human-specific insertions), 2) distinct from the remaining proviruses, and 3) proximal to, but not within, the 5’ LTR. We identified a highly conserved region of about 32 bp within the *gag* leader region just outside of the 5’ LTR and ~1 kb from the start of the HML-2 consensus sequence (Figure 1). BLAT searches for this sequence returned 25 hits, all of which corresponded to HML-2 proviruses; 17 were identical to the queried 32 bp sequence, and 8 had two or fewer mismatches (Figure 1). Of note, the matching sequences included all described human-specific proviruses that are present within the Hg19 build, thus providing further support for the specificity of the probe. We therefore took advantage of this sequence, referred to here as ‘*Kseq*’, for *in silico* restriction fragment analyses and subsequent DNA hybridizations to facilitate detection of the most conserved HML-2 proviruses present within our sampled genomes.

**Figure 2:**
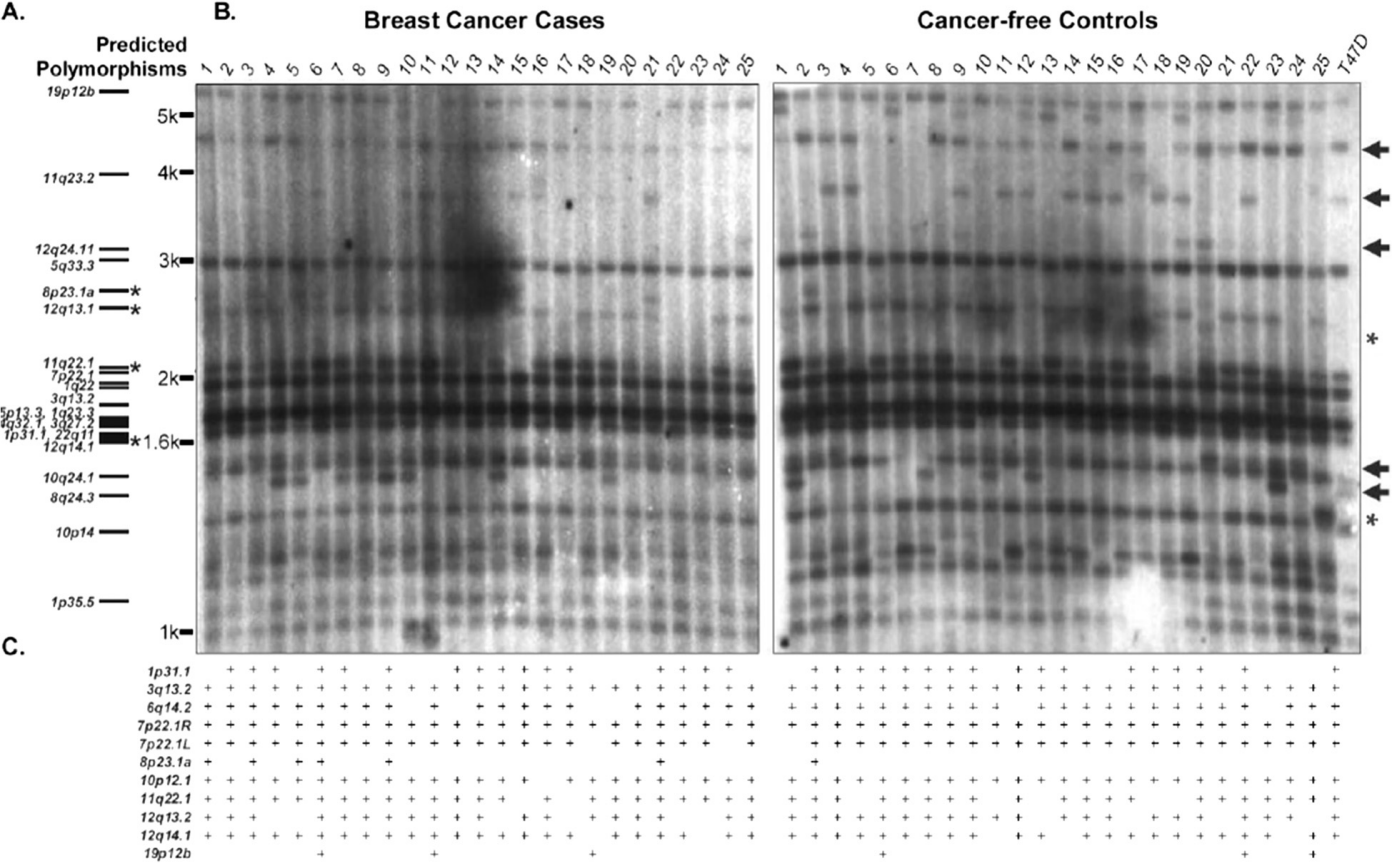
**Distribution of polymorphic HML-2 proviruses in breast cancer cases and controls. A**. Comparative schematic representing the *in silico*-predicted sizes for HML-2 containing fragments following *Bsr*I digestion and detected by the K-seq probe within the Hg19 genome build. Asterisks at left indicate the confirmed polymorphic proviruses, whose distribution coincides exactly between unblot banding patterns and PCR data. **B**. CPSII samples were sorted by case/control status (n = 25 each) and *Bsr*I digested WGA-DNA from each group was separated by gel electrophoresis and probed with the ^32^P-radiolabeled K-seq oligonucleotide. HML-2 junction fragments were visualized following exposure to film, and polymorphic insertions inferred by variable banding patterns among samples. **C**. Results from PCR analysis of known polymorphic proviruses for direct comparison of described polymorphic elements, where ‘+’ indicates the confirmed presence of the tested provirus. Novel polymorphic fragments whose identity could not be inferred by comparison to PCR analysis or *in silico* predictions, have been indicated with arrows at right. Asterisks (at right) are used to indicate the observed fragment sizes of polymorphic elements detected in ≤5% individuals screened here.

An *in silico* restriction analysis for the Hg19 human reference genome build was performed to identify candidate restriction enzymes for hybridization of the *Kseq* site within sampled human genomes. Each of the 25 elements identified by BLAT of the *Kseq* region was simultaneously analyzed for enzymes predicted to cut at least once within the provirus but not within the 5’LTR, as well as for the nearest restriction site within the host flanking DNA. As a result, each *Kseq*-containing ‘fragment’ is predicted to contain a single proviral junction site, whereas the size of each fragment is defined by the distance from the first cleavage site 3’ of the probe site to the nearest restriction site in host DNA (Figure 1, *upper*). Of about 35 candidate enzymes, 6 were analyzed in preliminary unblot screens using DNA from the T47D breast tumor-derived cell line (data not shown), and *Bsr*I was finally selected for further hybridization screening with reference to overall fragment size distributions (ranging from ~1 kb- > 6 kb) and total number of fragments predicted to contain HML-2 proviral junction sites (as many as 30; discussed further below). The *Bsr*I fragment distribution, as predicted from the Hg19 human reference build, is shown for reference in Figure 2A.

### Case–control analysis of polymorphic HML-2 proviruses in breast cancer

Based on *in silico* predictions, we utilized unblotting [14,58] to infer the distribution of polymorphic HML-2 proviruses within the genomes of the CPSII subjects. The unblotting technique is similar to Southern blotting DNA hybridization, but omits the transfer step of the DNA template, and consequently offers increased resolution without the loss of target DNA. A caveat is that at least 10 μg of template is required per sample per run, thus challenging the examination under conditions of limited quantities of genomic DNA, as was for the CPS-II samples (~1 μg per sample). We therefore subjected each sample to whole genome amplification (WGA; REPLI-g MIDI kit, Qiagen) step to generate working amounts of DNA per sample (at least 15 μg in our hands). The amplified samples were individually digested with *Bsr*I, and the products separated by electrophoresis through agarose. Simultaneous treatment of genomic DNA extracted from the T47D breast tumor-derived cell line was used as a control. The agarose was then dehydrated and the immobilized DNA hybridized with a ^32^P-labeled oligonucleotide complementary to the *Kseq* sequence, and finally exposed to film to visualize the prevalence and distribution of detectable HML-2-containing fragments. As with the initial HML-2-specific PCR screens of described polymorphic proviruses, all preliminary unblots were performed while samples were blinded, and following the subsequent release of their case/control status, the ublotting was repeated on a case–control basis and the samples analyzed by direct comparison between groups. Consistent with the cognate *in silico* analysis of the Hg19 human reference, unblots were interpreted such that each detected fragment represented a single proviral junction site, the size of which was dependent on the length of host sequence to the nearest 5’ flanking restriction site. The resulting unblots are shown by case/control group in Figure 2B.

Overall, the banding patterns we observed by unblot of WGA-DNA were in close agreement with those predicted by *in silico* restriction analysis of the Hg19 genome build (Figure 2A and B), implying uniform amplification of all regions of the DNA. On average, we observed between 18 and 22 bands per lane in fragments that varied from sample to sample. To further characterize the fragments observed to be polymorphic among individuals, we compared the distribution of polymorphic HML-2 proviruses with that of previously described copies, as interpreted by PCR screen. Comparison between HML-2-derived unblotting and PCR data allowed for the provisional assignment of a few fragments based on shared distribution between each analysis, each of which was further supported in agreement with the corresponding *in silico* predicted sizes (asterisks in Figure 2A). Most clearly represented were the fragments predicted to represent the 11q22.1 5’LTR junction, with a band around 2.1 kb that corresponded well with the expected distribution across all samples as determined by PCR (Figure 2B). Also near the 1.7 kb size, hybridized fragments were 100% consistent with the *in silico* size prediction and PCR distribution of the 12q14.1 provirus. Finally, we observed fragments matching the PCR distribution and size predictions of the 12q13.2 and K115 proviral junctions around 2.3 kb and 2.5 kb, respectively. At least two of the hybridized fragments (located at 11q22.1 and 12q13.2) could be unambiguously assigned to the corresponding HML-2 provirus by locus-specific amplification and sequencing of their 5’LTRs and host flanking regions from template DNA obtained by elution from the corresponding unblotted gel regions (data not shown).

For the remaining known polymorphic HML-2 proviruses, discrimination of their specific locus was less clear by comparison with previous PCR analysis in conjunction with *in silico* predictions. A few such elements were fixed or nearly fixed within the sample set as indicated by locus-specific PCR, for example the proviruses located at 3q13.2 and 7p22.1b, thus complicating their assignment, however all CPSII samples were observed to have hybridized fragments near the predicted sizes of these elements (respectively 1985 bp and 1981 bp; also refer to Table 2). The expected banding patterns for the 1p31.1 and 6q14.1 elements could not be discerned by 5’ LTR amplification, although the predicted junction fragments are around 1.7 kb (1751 for 1p31.1 and 1758 for 6q14.1). Given the number of bands that were both predicted and observed to fall within approximately the same size range, their specific banding patterns are likely to have been obscured. Another possibility is that some provirus-containing fragments may have been ‘lost’ due to the variable presence of common sequence polymorphisms within a meaningful *Bsr*1 site, or from sample-specific genomic structural variation in regions associated with HML-2 insertions; either scenario could potentially result in a junction fragment of an unexpected or undetectable size. Although this possibility cannot be excluded, we note the remaining predicted polymorphic HML-2 proviruses were consistent and well-supported among all results from unblotting, PCR screening, and *in silico* restriction analysis.

To identify putatively novel integration sites, we examined each unblot for polymorphic bands that were neither predicted by *in silico* analysis of the described HML-2 polymorphic proviruses within the available databases, nor consistent with any distribution observed by direct PCR analysis. Several fragments, with estimated sizes from 1.4 to 4.6 kb, were identified that met these criteria; these particular HML-2-containing fragments were clearly visible within multiple samples from either group, varying in frequency from ~0.02 to 0.98. One such band of interest, specifically in lane 25 of the control group at ~1.4 kb, was represented by a single band not observed in any other sample, whereas the opposite was observed for other fragments, for example the band visible around ~5.5 kb, which was present in the majority of samples (each example is indicated by an asterisk near the relative fragment size in Figure 2B, right). In all, roughly 5–10 polymorphic bands were visible, and of those, about 5 were clearly discernable across the total CPSII sample set (indicated in Figure 2B by arrows at right) The individual frequencies for each such fragment were directly compared between cases and controls by χ^2^ analysis (Table 4). Consistent with the initial PCR based analysis of described polymorphic elements as described above, no observed fragment differed significantly in its distribution between groups. The results indicate that at least within this sample set, polymorphic HML-2 proviruses do not imply association of a risk of breast cancer. However, our results also draw attention to an unexpected level of HML-2 content among these relatively few genomes tested in the present analysis.

**Table 4:** **Inferred case–control frequencies of previously undescribed polymorphic HML-2 proviruses in breast cancer**

**Observed band (bp)** ^***a***^	**Cases** ^***b***^		**Controls** ^***b***^			
	**# Positive**	**Frequency**	**# Positive**	**Frequency**	**χ** ^**2**^	***p*** **-value**
*4600*	25	1.00	22	0.88	3.19	0.07
*3700*	10	0.40	11	0.44	0.08	0.78
*3200*	1	0.04	4	0.16	2.00	0.16
*1500*	25	1.00	23	0.92	2.08	0.15
*1470*	8	0.32	5	0.20	0.93	0.33

^*a*^Band sizes are based on estimated fragment lengths; each has been indicated by arrow in Figure 2.

^*b*^Total sample size was 50 (n = 25 per group).

## Discussion

A few endogenous proviruses are known as causative to disease in experimental animal model systems, including the Betaretrovirus MMTV and mammary carcinoma in mice [15–20]. A similar association of the HML-2 proviruses, closely related to MMTV, is yet to be established, and remains a topic of study in the field. Here, we present our analysis of the distribution and prevalence of polymorphic HML-2 proviruses within the genomes of subsequently diagnosed breast cancer patients and from individuals with no history of the disease. For these purposes, we utilized two complementary approaches. We first developed a locus-specific PCR strategy to determine and assess the prevalence of each currently annotated polymorphic HML-2 locus with reference to the human reference database, as well as detection of the cognate unoccupied pre-integration sites and/or solo-LTR, where applicable. Secondly, we utilized unblotting, a high resolution and highly sequence-specific genome hybridization technique, as a means to provide direct inference of the prevalence and group distribution of putatively novel HML-2 polymorphic proviruses among the sampled genomes. For such proviruses, virtually nothing is known in terms of integration site, proviral structure, or functional features. To out knowledge, this is the first and most thorough report of such a comparison, and by far the largest representative set of human genomes analyzed for uncharacterized polymorphic proviruses from the most recently active HERV group.

The K113 and K115 proviruses were the first polymorphic HML-2 members to be discovered for which the empty-pre-integration site was still present within the population, and for which the proviral alleles were at relatively low frequencies, implying relatively recent germline integration (roughly estimated at <200,000 years and ~1.2 mya, respectively) [20]. In multiple reports, specific attention has been given to these two proviruses as possible candidates for roles in human diseases, including breast cancer [54], multiple sclerosis [56,57], schizophrenia [60], and autoimmune diseases [55,57]. Two of these reports are worth noting, in the context of the results presented here. In 2005, Moyes *et al*. [57] reported a “significantly” higher prevalence of the K113 provirus in the genomes of 109 multiple sclerosis patients. However, the analysis included multiple comparisons in terms of both proviruses tested and number of disease states, and the association was not replicated in a larger scaled study specifically addressing K113 prevalence and multiple sclerosis [56], highlighting the importance of being able to test such an initial finding on a statistically supported scale. Also pertinent is the 2004 report from Burmeister *et al*., in which K113 was observed at a somewhat higher frequency in individual breast cancer patients from an initial screen of 102 patients’ genomes [54]. This particular result lacked statistical significance and was not further tested in larger screens. In the present study, our initial observation of a higher prevalence of the K115 provirus to breast cancer cases was not replicated in an independent set of samples, which we were fortunate to have been made available to us through the ACS CPSII Nutrition Cohort Study. Given the negative outcome of the PCR analysis of the second, larger sample set, the necessity for such added analysis is made clear.

In previous investigations for evidence of disease association, frequencies of the K113 and K115 proviruses have been reported to range from ~10-20% for K113 and ~5-12% for K115 [20]. Our results are consistent with these observations, with the exception of the K115 provirus in ~24% of cases in the initial screen (Figure 2B and C). This frequency is not completely unexpected, however, as values as high as >40% have been reported, depending on the race of the samples tested [20,57]. Similarly, in other analyses the K113 provirus has been observed at levels as high as ~30%, again depending on race [20,57]. Given such variance, the observed frequencies of the K115 provirus among DNAs from breast cancer cases may reflect an uneven representation with regards to ethnicity in the sample set. Alternatively, the higher frequency of K115 we observed in cases could be due to stochastic effects from the relatively small sample size used for the present analysis. As the samples were de-identified, we can only speculate on the factors, if any, influencing the observed distribution.

To date, all reports that have attempted to detect a genetic association of individual HML-2 proviruses have had a predominant focus to K113 and K115, given their status as the most recently integrated and conserved HML-2 loci known, however their analysis (over several diverse populations and disease groups) have offered little support for any implications in disease. This is perhaps not surprising, as a provirus that did have negative effects to the host would have a much reduced probability of population fixation, or would likely be removed from the population by selection. Thus, those proviruses with rare frequencies among humans would be more appropriate candidates for inference of disease-associated loci. The detection of such elements will necessitate much larger sample sizes than have been used to date, including the analysis presented here. Repeated searches for a disease association with one or two particular elements alone, such as has been the case for the K113 and K115 proviruses, will likely have similar outcomes as have been observed. We attempted to overcome such limitations by screening human genomes from the CPS-II ACS Nutritional Cohort using a highly specific DNA hybridization in a case-control comparison; we interpret our data to indicate the detectable presence of several as-yet-uncharacterized polymorphic proviruses, though none infer genetic association to disease.

We note that, although “new” bands observed from the unblots have a high likelihood of representing HML-2 containing genomic fragments, they may not reflect previously undescribed proviruses. For example, they could possibly have been a consequence of single base changes in known proviruses that destroyed or created a target sequence for *Bsr*I restriction enzyme cleavage, Furthermore, the absence of certain bands in some samples could result from known full-length proviruses that have recombined to form solo LTRs in some individuals, or from recombination-mediated structural variation with reference to the human Hg19 build that would be undetectable in our approach. Also, a point mutation could lead to the generation of a new restriction site, for example within the 5’ LTR, that would prevent the detection of the corresponding junction fragment by the probe. We searched for such an example from the fragments that we could tentatively identify as described HML-2 (asterisks in Figure 2A), and found the PCR and unblot data were in agreement, giving support that the *Bsr*I target sites for these particular elements have not been disrupted. However, without knowledge of the chromosomal site of integration for each detected fragment, it is difficult to exclude the possibility of mutation (or possibly common SNPs among subjects) having occurred at restriction sites proximal to other detected proviruses.

In this study, we have developed an approach to identifying novel polymorphic proviruses in human populations, starting with samples of nanogram quantities of DNA, and we have provided evidence for a number of polymorphic proviruses that vary in frequency among the samples tested, some of which are present at quite low frequencies (for example, in lane 25 of the ‘undiagnosed controls’ in Figure 2B, asterisk at right). For the ~50 genomic DNAs in this analysis, between 18 and 22 bands were observed per sample. In the total set, there were about 10-15 junction fragments for which a corresponding known provirus could not be inferred from comparison to *in silico* or PCR analyses. Given the sample size, it is likely that at least some of these HML-2-containing fragments represent recent bona fide proviral integrations, which are present in just a portion of individuals, as would be predicted for such sites. At least two fragments, of sizes around 2.2 kb (in undiagnosed controls, sample 20) and 1.3 kb (same group, sample 25) (also asterisked in Figure 2B, right) appear to be present in less than ~5% of the total number of samples –a far lower representation than seen for any other described polymorphic provirus or previous report [16,17]. If not represented by solo LTRs in other individuals, such a provirus is likely to have been recently integrated and to closely resemble the original infecting virus, and, we can speculate, might also exhibit retained competency for replication. Current and future efforts to identify and characterize such novel proviruses will likely help in clarification of disease and/or phenotypic association of such sites.

## Conclusions

In this study, we investigated the possible relationship between the genome-wide presence of polymorphic HML-2 proviruses in 50 humans with regard to breast cancer diagnosis. Although preliminary PCR analysis indicated the possibility of an elevated prevalence of one particular provirus, K115 (located at 8p23.1), in cases compared to controls and supported in in DNA hybridization screening, the observation was not replicated to a statistically significant level. Thus, we find no difference in the prevalence of proviruses between groups, suggesting that common polymorphic HML-2 proviruses are not associated with breast cancer in the tested individuals. These findings do not exclude either the possibility that rare HML-2 proviruses are involved in a subset of breast cancers, or their possible utility as tissue-specific expression and/or HML-2-derived products as potential marker(s) of disease. Interestingly, our findings do indicate a relatively high level of putatively novel HML-2 sites within the population, providing support for additional relatively recent insertion events and implication for ongoing activities. With continued improvements in sequencing technologies and in the detection of such elements, it is likely novel HML-2 polymorphic loci will be identified in the near-future; their genome-wide implications in terms of population-level structural variation and/or outcome phenotypic effects will remain, until then, to be seen.

## Methods

### Human DNA samples

Human genomic DNA samples were from the ACS Cancer Prevention Study II Nutrition Cohort (CPS-II), a prospective study of cancer incidence of approximately 184,000 Americans [59]. Nutrition Cohort participants, who were from 21 states and ranged from 50 to 74 years old at enrollment in 1992 or 1993, completed a mailed questionnaire that included questions on demographics, diet, and other lifestyle factors. Incident cases reported via questionnaire response were verified through medical records, linkage with state cancer registries, or death certificates. Blood samples were collected from a subset of Nutrition Cohort participants (21,965 women and 17,411 men) between June 1998 and June 2001, fractionated and stored in liquid nitrogen vapor phase at −130°C until needed for analysis. All aspects of the CPS-II Nutrition Cohort study were approved by the Emory University Institutional Review Board (Atlanta, GA). Original CPS-II samples provided by the ACS were 100 total: 50 samples were from participants who were later diagnosed with breast cancer, and controls (n = 25 per group); 50 samples were from participants who were later diagnosed with prostate cancer, and controls (also 25 per group). Controls were from participants who were cancer free at the time of diagnosis of the matching case. Samples were blinded, and subsequently unblinded following initial PCR analyses. All samples were deidentified, with information limited to case/control assignment. To account for multiple comparisons, secondary PCR screens were performed with an additional 200 genomic DNA samples from the CPS-II cohort (n = 100 per breast cancer cases or controls). As above, all samples were deidentified, and case/control information unblinded following PCR screening.

### Whole genome amplification

To obtain sufficient DNA for unblotting and PCR analyses, individually screened CPS-II DNA samples (~1 μg) were subjected to whole genome amplification (WGA). WGA was carried out according the manufacturer’s protocol (MIDI Repli-G, Qiagen) with a starting volume of 5 μL. Briefly, ~40 ng genomic DNA per sample was denatured and neutralized using the supplied buffers in volumes of 5 μL and 10 μL, respectively, for 3 min each at room temperature (RT). A mixture containing buffered φ29 polymerase (MIDI Repli-G, Qiagen) and random hexamers was added to each sample for a final volume of 50 μL and the samples incubated 16 hr. at 30°C. Amplified DNA was extracted using 2 mL heavy phase-lock gel tubes (5 PRIME) in 200 μL volumes according to the manufacturer’s protocol. DNA was precipitated from the aqueous phase in 95% ethanol + 100 mM NaOAc, pH 5.2 to a final volume of 1 mL and incubated overnight at -20°C. The WGA DNA was pelleted at 14,000 rpm for 30 min. at 4°C, washed in 1 ml cold 70% ethanol, the centrifugation repeated, and the ethanol carefully aspirated. Pellets were dried 30 min. at 37°C, resuspended in 100 μL sterile water, and the WGA DNA measured using a NanoDrop spectrophotometer.

### PCR amplification

For 11 loci with evidence of multiple alleles including the provirus form, locus-specific primers were designed to amplify the 5’ LTR of the provirus at each site using the most recently updated human genome Hg19 reference build (Table 1). For each locus, a primer was designed within ~2 kb of the provirus edge within the flanking DNA of the host, and a second primer within the proviral leader sequence, outside of, but near, the 5’ LTR. A third primer was designed in the host DNA downstream of the integration site in order to detect and differentiate the remaining alleles, including solo LTRs and unoccupied integration sites. Primers were designed using Primer3 v.0.4.0 and obtained from IDT, unless otherwise noted. An *in silico* PCR (UCSC Genome Browser) was used to estimate target amplification and product size for each primer pair, as provided in Table 1. All PCRs were carried out using ~200 ng WGA DNA as template with 1.5-2.5 μM Mg^++^, 200 μM dNTPs, 0.2 μM each primer, and 2.5 U Platinum Taq Polymerase (Invitrogen). 10 uL of each PCR reaction were analyzed by electrophoresis through 1% agarose in 1 × TBE. Products from 2 separate positive PCR reactions per primer set were sequenced to confirm the desired product.

### *In silico* restriction analysis

We used an *in silico* approach to identify useful restriction enzymes for subsequent DNA hybridizations to visualize HML-2 proviruses, and to generate a restriction fragment comparison from existing genome sequence data for reference during unblotting (see below). The HERV-K113 sequence (AY037928) was analyzed for restriction enzymes predicted to cut at least once within the provirus but not within the 5’LTR (NEBCutter2.0), for a total of 36 candidate enzymes. Simultaneously, we mined the 2009 human genome build (GRCh37/hg19) for proviruses with high percent identity to HML-2, again using the K113 nucleotide sequence as a reference. For the 32 proviruses identified from the search, we performed an *in silico* restriction analysis as follows. About 5 kb of sequence was extracted in both directions from the start of the 5’LTR. Each sequence was ‘digested’ in NEBCutterV2.0 for each of the 36 restriction sites with reference to a highly conserved sequence spanning bases 1017 to 1049 (5’ CGTCGACTTCTTGTCCTCAATGACCACGC; HERVK-1017). For each enzyme analyzed, the estimated sizes for predicted HERV-K-containing junction fragments were plotted on a log scale for comparison. Based on restriction fragment estimates with reference to genome coverage and the observed fragment distribution, *Bsr*I was selected for unblot analysis and coordinate *in silico* comparison to the published sequence.

### Unblotting

Unblotting, or hybridization in semi-dried agarose [14,58], was carried out to visualize polymorphic HERV-K proviruses within DNA samples. For each sample, 15 μg of WGA DNA was digested with *Bsr*I (New England Biolabs) in a 100 μL volume and the digested products extracted and precipitated as described above. Products were resuspended in 20uL 0.25 x TBE + 30% Ficol and electrophoresed through a 0.8% agarose gel in 0.25 × TBE at 70 V for 29 hr. at 4°C. The gel was dehydrated in a vacuum dryer (BioRad) layered on filter papers for 60 min. at RT and 60 min. at 62°C. The dried gel was stained with ethidium bromide in 0.25× TBE and excess agarose removed with a clean scalpel. The gel was then incubated in denaturing buffer (0.5 M NaOH + 1.5 M NaCl), and neutralizing buffer (1.0 M Tris–HCl + 1.5 M NaCl, pH 8.0) 30 min. each at RT, and then hybridized with 7.5 × 10^6^ cpm of a ^32^P-labeled HERVK-1017 HML-2-specific oligonucleotide. Hybridization was in 5 mL of 5× SSPE (3.0 M NaCl, 0.2 M NaH_2_PO_4_, and 0.02 M EDTA, pH 7.4) + 0.1% SDS, pH 7.4 at 53°C for 16 hr with shaking at 50 rpm. Following hybridization, the gel was washed (2× SSC + 0.1% SDS) 4× for 15 min. each at RT, and 2× for 30 min. each at 53°C with shaking at 70 rpm. The gel was then exposed to BioMax MS film (Kodak) under an intensifying screen for 4–5 days at −70°C.

### Statistical analyses

Frequencies of individual sites were analyzed between case/control groups by χ^2^ analysis with one degree of freedom. For these analyses, comparisons were between cases and controls for individual polymorphic proviruses, calculated from 50 total samples (25 breast cancer per group). A *p* value of less than 0.05 was taken to be significant. Total numbers of samples for scaled screening were determined by power analysis. For K115, to replicate a 20% difference between test groups with an α = 0.05, a statistical level of 80% power requires a sample size of n = 94 (47 per cases and controls), and for 90% power a total sample size of n = 124 (62 each group). All statistical analyses were performed by the Data Design and Resource Center at Tufts University.

## Abbreviations

ACS: American Cancer Society
BLAT: BLAST-like alignment tool
CPSII: ACS Cancer Prevention II Nutritional Cohort
DNA: Deoxyribonucleic acid
ERV: Endogenous retrovirus
GWAS: Genome-wide association study
HERV: Human endogenous retrovirus
HML-2: Human MMTV-like
JSRV: Jaagsietke Sheep Retrovirus
LTR: Long terminal repeat
MMTV: Mouse Mammary Tumor Virus
ORF: Open reading frame
PCR: Polymerase chain reaction
RVLP: Retrovirus-like particle
SNP: Single nucleotide polymorphism
WGA: Whole genome amplification
